# Demonstration of thermal modulation using nanoscale and microscale structures for ultralarge pixel array photothermal transducers

**DOI:** 10.1038/s41378-021-00315-5

**Published:** 2021-12-03

**Authors:** Jinying Zhang, Defang Li, Zhuo Li, Xin Wang, Suhui Yang

**Affiliations:** 1 Beijing Key Laboratory for Precision Optoelectronic Measurement Instrument and Technology, School of Optics and Photonics, Beijing Institute of Technology, 100081 Beijing, P. R. China; 2 Yangtze Delta Region Academy of Beijing Institute of Technology, 314001 Jiaxing, P. R. China; 3 Institute of Semiconductors, Chinese Academy of Sciences, 100083 Beijing, P. R. China

**Keywords:** Sensors, Optical sensors, NEMS

## Abstract

Large-pixel-array infrared emitters are attractive in the applications of infrared imaging and detection. However, the array scale has been restricted in traditional technologies. Here, we demonstrated a light-driven photothermal transduction approach for an ultralarge pixel array infrared emitter. A metal-black coating with nanoporous structures and a silicon (Si) layer with microgap structures were proposed to manage the thermal input and output issues. The effects of the nanoscale structures in the black coating and microscale structures in the Si layer were investigated. Remarkable thermal modulation could be obtained by adjusting the nanoscale and microscale structures. The measured stationary and transient results of the fabricated photothermal transducers agreed well with the simulated results. From the input view, due to its wide spectrum and high absorption, the black coating with nanoscale structures contributed to a 5.6-fold increase in the temperature difference compared to that without the black coating. From the output view, the microgap structures in the Si layer eliminated the in-plane thermal crosstalk. The temperature difference was increased by 340% by modulating the out-of-plane microstructures. The proposed photothermal transducer had a rising time of 0.95 ms and a falling time of 0.53 ms, ensuring a fast time response. This method is compatible with low-cost and mass manufacturing and has promising potential to achieve ultralarge-array pixels beyond ten million.

## Introduction

Infrared emitters have been widely used in infrared imaging and detection fields^1–3^. They are core devices in many applications such as infrared scene generation technology. Numerous researchers have contributed to the development of infrared emitters and achieved very large performance advances in terms of, for example, array scale, temperature contrast ratio, and time response^4–6^. Several typical methods have been utilized, including digital micromirror devices (DMDs), resistor arrays, light-emitting diodes (LEDs) and microelectromechanical system (MEMS) thin-film emitters^7–12^. For the DMDs, the maximum pixel array is 1920 × 1080, the apparent temperature is more than 800 K, and the 8-bit video frame rate reaches 690 Hz^13^. For the resistor arrays, the pixel array reaches 1536 × 768, the maximum apparent temperature of the infrared mid-wave band is 675 K, and the frame rate reaches 200 Hz^5^. For the LEDs, the maximum pixel array is 512 × 512, the apparent temperature is as high as 1800 K, and the rising time reaches the microsecond level^14,15^. However, the fabrication of these devices is complicated, and the layout of read-in or read-out integrated circuits becomes difficult with increasing array scale^16^. MEMS thin film emitters are based on light-driven technology and do not require integrated circuits^4^. In recent years, great progress has been made in the properties of MEMS thin-film infrared emitters^17–20^. Researchers have used a two-dimensional (2D) suspended thin film to transform visible light energy into infrared rays^20^. These MEMS thin-film infrared emitters utilized a metal-black coating prepared by thermal deposition as the absorption layer. Compared to other high light-absorption materials (such as carbon nanotubes^21^ and black silicon^22^), metal-black coatings have the advantages of low density and low heat capacity, which contribute to a better time response for infrared emitters. In addition, due to the mature processing technology of infrared emitters, the pixel array achieved was sized 1300 × 1300, the apparent temperature was more than 489 K, and the frame rate was 50 Hz^23^. However, the 2D MEMS thin film emitter utilized in-plane microstructures to control the thermophysical properties^17,18^. It could not control the out-of-plane thermal diffusion, resulting in slow heat dissipation. Consequently, it was challenging to further increase the frame rate. The pixels of the infrared emitter were connected to adjacent pixels, so there was still a thermal crosstalk problem, and the temperature contrast ratio was constrained^19,20^. Moreover, limited to the thickness of 2D thin films, the poor mechanical stability and larger array scale of the infrared emitter has become a challenge^24^.

A robust infrared emitter with a greater heat dissipation rate and mechanical stability was proposed and investigated by our group. Additionally, the array scale was enlarged to 2000 × 2000^24^. We introduced silicon (Si) microcavities to isolate the pixels and the Si substrate to maintain a relatively high-temperature contrast ratio. However, the thermal diffusion through the pixel frames contacting the Si substrate was too fast to obtain a more desirable contrast ratio. Therefore, the thermal management issue urgently needed to be addressed to achieve superior performance enhancement.

In this work, we proposed a metal-black coating with nanoporous structures and a Si layer with microgap structures to manage the input and output thermal issues. Based on a light-driven photothermal transducing approach, we demonstrated an ultralarge pixel array infrared emitter. The effects of the nanoscale structures in the black coating and microscale structures in the Si layer were investigated. Remarkable thermal modulation could be obtained by adjusting the nanoscale and microscale structures. The measured stationary and transient results of the fabricated photothermal transducers were in good agreement with the simulated results. The black coating with nanoscale structures contributed to a 5.6-fold increase in the temperature difference compared to that without the black coating. The in-plane thermal crosstalk was eliminated. The temperature difference was increased by 340% by modulating the out-of-plane microstructures. The proposed photothermal transducer had a rising time of 0.95 ms and a falling time of 0.53 ms, ensuring a fast time response. This modulation strategy capitalized on nanoscale and microscale structures was important for optimizing the stationary and transient thermal properties for photothermal transducers, and provided good guidance for other MEMS devices that required thermal management. In particular, the mechanical stability of the proposed photothermal transducers was advantageous, and the manufacturing method was quite simple and low-cost and had great potential to achieve a much larger pixel scale provided that larger Si processing was guaranteed.

## Structures of the photothermal transducer

**Fig. 1:**
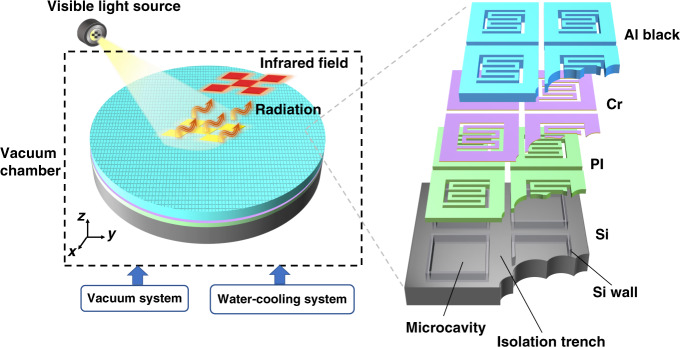
Schematic of the photothermal transducer driven by visible light. Working principle of the photothermal transducer (left) and structure of each pixel (right). Al aluminum, Cr chromium, PI polyimide, Si silicon

The photothermal transducer was driven by visible light. The pixels absorbed the light energy, and their temperature increased to emit the corresponding infrared ray according to Plank’s blackbody radiation principle^25^. To eliminate air convection and keep the substrate at a constant temperature (e.g., 300 K), the photothermal transducer was clasped in a vacuum chamber connected with a vacuum system and a water-cooling system. Figure 1 illustrates a schematic of the photothermal transducer driven by visible light. The transducer consisted of four layers. From top to bottom, they were an aluminum (Al) black coating layer, a chromium (Cr) layer, a polyimide (PI) layer and a Si layer.

The thermal input and output issues of the photothermal transducer could be managed by modulating the nanoporous structures in the Al black coating and the microgap structures in the Si layer.

The Al black coating contained nanoporous structures and possessed strong visible light-absorption and infrared ray emission abilities^24,26^. As presented in Fig. S1 (see supplemental Section S1), the fabricated black coating showed cluster structures full of nanopores on the scale of tens of nanometers and nanogaps between clusters on the scale of hundreds of nanometers. These nanoscale structures ensured ultrabroadband absorption in the visible range, and the measured absorption was beyond 90%. Incident photons were trapped in the black coating. They experienced multiple scattering events in these nanopores and nanogaps and hit microscopic particles. The particles absorbed the photons’ energy, their thermal motion was enhanced, and the thermal energy was increased. Consequently, the input of the photothermal transducer was attributed to the absorption ability of the black coating. The absorption ability could be modulated by changing the thickness of the coating and the dimensions of the nanopores and nanogaps. As illustrated in Fig. S2 (see supplemental Section S1), the Al black coating with nanoscale structures contributed to a 5.6-fold increase in the temperature difference compared to that without Al black coating.

Suspended on the Si microcavities, the PI layer served as a supporting layer with low thermal conductivity to accumulate thermal energy^27^. The Cr layer was an adhesive thin film with a thickness of only several nanometers. This contributed to enhancing the combination of the Al black coating and the PI layer.

The Si layer contained microgap structures including microcavities and micro-isolation trenches. Si walls were formed between the microcavities and the isolation trenches. The microcavities were used to isolate the pixels and the Si substrate to obtain a relatively high temperature. The isolation trenches played an important role in both in-plane and out-of-plane thermal modulation. On the one hand, the in-plane thermal diffusion between adjacent pixels was isolated to eliminate thermal crosstalk. On the other hand, the Si walls became thinner when the isolation trenches were wider. Thus, the thermal resistance of the Si walls increased, and the out-of-plane thermal diffusion rate decreased. For a single pixel suspended on the Si cavity and isolated with adjacent pixels by the isolation trenches, there was only out-of-plane heat conduction and no in-plane conduction. Therefore, the process could be simplified as one-dimensional heat transfer along the *z* (out-of-plane) direction of the Si wall. The thermal resistance could be calculated using

1 $$R_{{{{\mathrm{cond}}}}} = \frac{{T_{s,1} - T_{s,2}}}{{q_z}} = \frac{H}{{kA}}$$

where *R*_cond_ is the thermal resistance of the Si wall, *T*_s,1_ and *T*_s,2_ are the temperatures on each side of the Si wall, and *q*_z_ is the heat conduction rate along the *z* axis. *H* is the height of the Si wall. *A* is the cross-sectional area perpendicular to the direction of heat flux. In addition, *A* is proportional to the width of the Si wall. *k* is the thermal conductivity of the Si material.

Evidently, the thermal resistance is inversely proportional to the width of the Si wall, so we could adjust the out-of-plane thermal diffusion rate by changing the width of the Si wall. Note that the thermal resistance is inversely proportional to the thermal conductivity of the Si material (*k*). This material parameter has a size-dependent effect. When the dimension of the Si structures is comparable to or smaller than the mean free path (MFP) of phonons in Si material (approximately 43 nm^28^), the value of *k* is significantly different from the bulk Si material value^29^. Considering that the fabrication of such small structures would be extremely difficult and that the mechanical stability cannot be guaranteed, we chose to set the critical features of the Si wall to the micron level, far from the MFP of the Si material. Therefore, the cross-sectional area *A* determined the value of the thermal resistance *R*_cond_.

Different from the traditional 2D modulation method, the proposed photothermal transducer capitalized on a 3D thermal management strategy based on in-plane and out-of-plane microstructures. The influence of the out-of-plane microstructures in the Si layer and in-plane patterns in the pixels was investigated using finite element simulation and measurement experiments.

## Finite element simulation

### Effect of out-of-plane structures

Systematic numerical simulations were carried out based on the finite element method (FEM). First, to compare the thermal modulation abilities of the traditional 2D in-plane approach and the proposed 3D strategy, we modeled 2D and 3D photothermal transducers. Considering the important role of the isolation trench in the Si layer, the 3D models included two types: a 3D model with isolation trenches (3D-IT) and a 3D model with no isolation trenches (3D-NIT).

The material parameters of the three structures were set to be the same, as shown in supplemental Section S2. In the FEM model, the temperature and radiation of the photothermal transducers were calculated by the following heat transfer formula:^28^

2 $$\rho dc_p\frac{{\partial T}}{{\partial {{{\mathrm{t}}}}}} = Q + kd\frac{{\partial ^2T}}{{\partial \overrightarrow {{{\mathrm{r}}}} ^2}} - \sigma \varepsilon \left( {T^4 - T_{{{{\mathrm{amb}}}}}^4} \right)$$

where *ρ*, *d*, *c*_*p*_, *k*, *ε* and *T* are the density, thickness, specific heat capacity, thermal conductivity, emissivity and temperature of the thin film, *Q* is the power density absorbed by the Al black coating, ${{{\vec{\mathrm r}}}}$ is the inner normal direction, *σ* is the Stefan-Boltzmann constant of 5.67 × 10^−8^ W/(m^2^ K^4^), and *T*_amb_ is the ambient temperature. The photothermal transducer usually worked in a high vacuum environment (<5 × 10^−4^ Pa), so convective heat transfer was neglected. The left term of the equation describes the transient temperature change of the model. The right term of the equation presents the absorption, conduction, and radiation of the model.

**Fig. 2:**
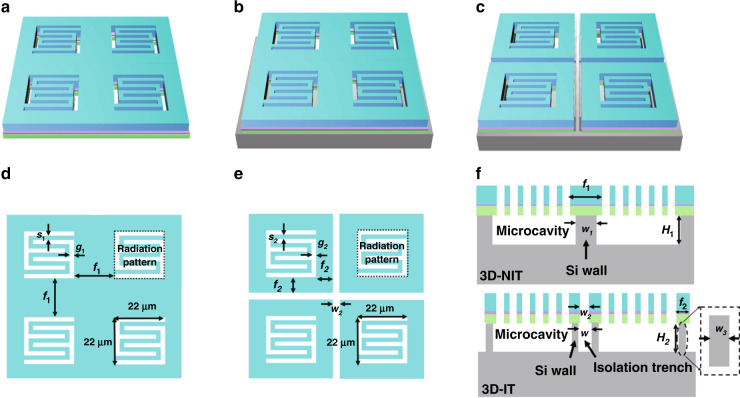
Structural diagrams of the 2D, 3D-NIT and 3D-IT transducer models. **a** 2D transducer model. **b** 3D-NIT transducer model. **c** 3D-IT transducer model. **d** In-plane patterns of the 2D and 3D-NIT transducer models. **e** In-plane pattern of the 3D-IT transducer model. **f** Out-of-plane structures of the 3D-NIT (top) and 3D-IT (bottom) transducer models

**Fig. 3:**
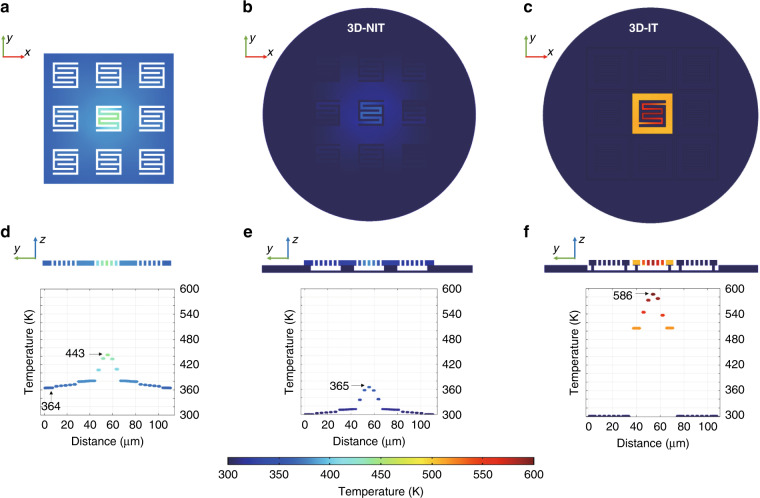
Stationary temperature distribution of the three models. **a** Top view of the 2D transducer model. **b** Top view of the 3D-NIT transducer model. **c** Top view of the 3D-IT transducer model. **d** Cross-sectional view of the 2D transducer model. **e** Cross-sectional view of the 3D-NIT transducer model. **f** Cross-sectional view of the 3D-IT transducer model

The structural parameters of the in-plane patterns are displayed in Fig. 2 for the three models. The 2D transducer (Fig. 2a) had no Si layer, and its in-plane patterns were identical to the 3D-NIT pattern, as displayed in Fig. 2d. The double-S strip was 2 μm wide (*s*_1_), the gap was 2 μm wide (*g*_1_), and the pixel frame was 15 μm wide (*f*_1_). The difference between the 3D-NIT (Fig. 2b) and 3D-IT (Fig. 2c) transducers concerns the isolation trench. As shown in Fig. 2e, in the 3D-IT transducers, the double-S strip was 2 μm wide (*s*_2_), the gap was 2 μm wide (*g*_2_), the pixel frame was 6 μm wide (*f*_2_) and the frame gap was 3 μm wide (*w*_2_). Since the isotropic etching process of Si was utilized in the fabrication of Si microcavities, there was a lateral undercut beneath the pixel frames. Thus, the Si wall was thinner than the pixel frame. As shown in Fig. 2f, the Si wall had a width of 11 μm (*w*_1_), smaller than *f*_1_ in the 3D-NIT model. In the 3D-IT model, the Si wall had a width of 2 μm (*w*_3_), smaller than *f*_2_, and the width of the trench (*w*) was 7 μm, larger than *w*_2_ because of isotropic etching. The height of the Si wall (*H*_1_ and *H*_2_) was approximately 5 μm.

The physical pixel array of the photothermal transducer was more than 2000 × 2000, which would result in extensive computational costs and memory use in the simulation. To reduce the calculation overhead, we set the pixel array to 3 × 3 and only applied light power to one pixel to observe its influence on the neighboring pixels.

In the 3D-NIT and 3D-IT transducer models, the temperature of the back surface of the Si substrate and the transducer’s initial temperature were set to 300 K. In the 2D transducer model, there was no substrate, and the initial temperature was 300 K. For the transducers with connected pixels (2D and 3D-NIT), the calculation demonstrated that the distance of lateral thermal conduction for a single powered pixel was larger than the length of three pixels. Therefore, infinite element fields were set at the four outer edges of the pixel array, and their boundary temperature was set to 300 K. Considering thermal radiation in vacuum, the top surface of the transducer had a surface emissivity of 0.80. A light source with a power density of 16 W/cm^2^ was applied to the single center pixel in the three models.

The temperature contrast ratio of the infrared imaging could be reflected from the stationary temperature difference. Figure 3 presents the simulated stationary temperature field distribution of the three models in a 3 × 3 pixel array.

Figure 3a–c displays the top view of the temperature field distribution for the three models. The cross-sectional views of the temperature field distributions for the three models are presented in Fig. 3d–f. The results indicated several points. First, the highest temperature of each model was located at the center of the powered pixel. Second, the pixels in the 2D and 3D-NIT transducers were connected to each other rather than independent, so thermal crosstalk was inevitable. Due to disconnection of the structures by the isolation trench, the crosstalk was eliminated between the adjacent pixels in the 3D-IT transducer. Third, the temperature differences for the 2D, 3D-NIT and 3D-IT models were 79 K, 65 K and 286 K, respectively. The thermal conduction in the 2D transducer was in-plane diffusion, which caused significant crosstalk to neighboring pixels. This resulted in a poor temperature contrast ratio. In the 3D transducers, the Si wall played an important role in out-of-plane heat dissipation through the pixel frame to the Si substrate. Thus, the Si wall was similar to a drainage pipe, and the crosstalk was significantly reduced. However, while the crosstalk of the 3D-NIT transducer decreased, the maximum temperature also declined. To enhance the temperature difference, isolation trenches were introduced to the 3D-NIT transducer. Finally, the temperature difference increased by approximately 340% from the 3D-NIT transducer to the proposed 3D-IT transducer.

In summary, the 3D-IT transducer exhibited the temperature contrast ratio properties superior to those of the 2D and 3D-NIT transducers. It also had mechanical stability advantages and great potential in ultralarge-scale arrays, so the following simulation study focused on 3D-IT transducers.

### Effect of in-plane patterns

**Fig. 4:**
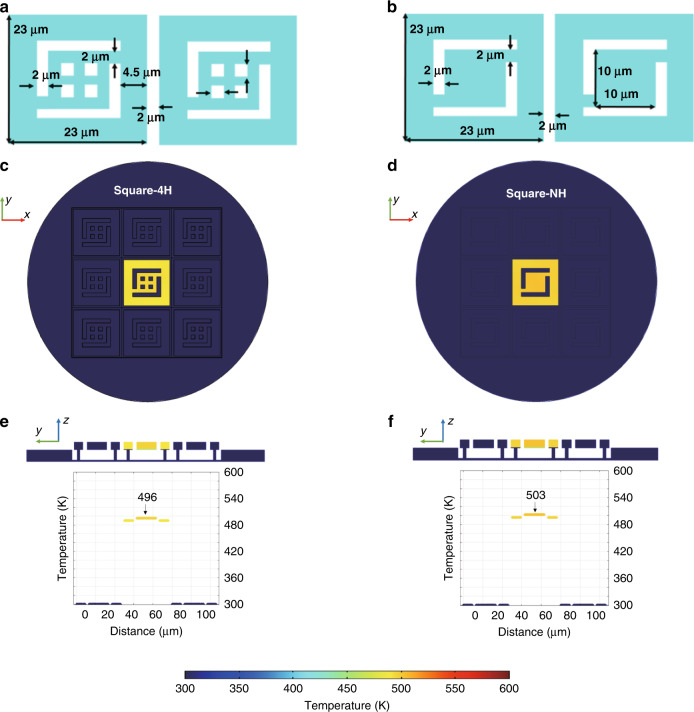
Schematics and simulated results of the Square-4H and Square-NH transducer models. **a** In-plane pattern of the Square-4H transducer model. **b** In-plane pattern of the Square-NH transducer model. The corresponding simulated stationary temperature fields: **c** Top view of the Square-4H transducer model. **d** Top view of the Square-NH transducer model. **e** Cross-sectional view of the Square-4H transducer model. **f** Cross-sectional view of the Square-NH transducer model

Three in-plane patterns were designed in the FEM simulation. The first pattern was a double-S (DS) strip pattern, as illustrated in Fig. 2e, and we named this pattern the DS transducer. The second is presented in Fig. 4a. The pixel pattern was a square with 4 holes (Square-4H) and was named the Square-4H transducer. The third pattern is presented in Fig. 4b. The pixel pattern was a square with no holes (Square-NH) and was named the Square-NH transducer. Their calculated stationary temperature fields are presented in Fig. 3c, f and Fig. 4c–f. As discussed above, the maximum temperature difference was 286 K for the DS transducer. Figure 4e, f shows that the maximum temperature differences were 196 K and 203 K for the Square-4H and Square-NH transducers, respectively.

Based on these results, we could obtain several conclusions. First, the Square-4H and Square-NH transducers had compatible temperature contrast ratios since the difference in their maximum temperature difference was only 7 K. The DS transducer had a temperature difference approximately 40% higher than those of the Square-4H and Square-NH transducers. Second, the DS and Square-4H transducers had a similar duty ratio of approximately 45%. This indicated they had the same light power absorption. The higher temperature difference of the DS transducer was attributed to the larger in-plane thermal resistance. In this case, heat tended to accumulate since the in-plane thermal diffusion was slow. Third, the Square-4H and Square-NH transducers had similar temperature differences, although they had different duty ratios. This was because the Square-NH transducer had a smaller in-plane thermal resistance, and consequently, its in-plane thermal diffusion was faster than that of the Square-4H transducer. As a result, the Square-NH transducer accepted more power, and its heat dissipation was faster, so it obtained a temperature difference comparable to that of the Square-4H transducer.

Notably, the in-plane thermal resistance was proportional to the length and inversely proportional to the cross-sectional area of the in-plane pattern. Evidently, the DS pattern had the largest thermal resistance because it had a larger length and smaller cross-sectional area than the Square-4H and Square-NH patterns. The in-plane patterns in the Square-4H and Square-NH transducers were complicated. The calculation of their in-plane thermal resistance was a complex 2D problem. Generally, the Square-NH transducer had a smaller equivalent thermal resistance because it had a higher duty ratio than the Square-4H transducer.

Considering that the DS transducer exhibited the most desired temperature contrast ratio, the following fabrication was carried out based on this pattern.

## Experiment and measurement

### Fabrication results

In the Si layer, the isolation trench played a key role in both in-plane and out-of-plane thermal diffusion. To investigate the effect of the 3D microstructures in the Si layer, we fabricated two kinds of transducers without and with isolation trenches. The proposed 3D-NIT and 3D-IT transducers with the same DS in-plane pattern were fabricated through spin-coating, deposition, lithography and etching processes that were compatible with integrated circuit (IC) mass production technology (see supplemental Section S3). We fabricated a 3D-NIT transducer with pixels exceeding 2000 × 2000, and the testing results demonstrated that the load weight was beyond 1700 g^24^. Since the 3D-IT transducer had structures and fabrication methods similar to those of the 3D-NIT transducer, it would still be advantageous in array scale and mechanical stability.

**Fig. 5:**
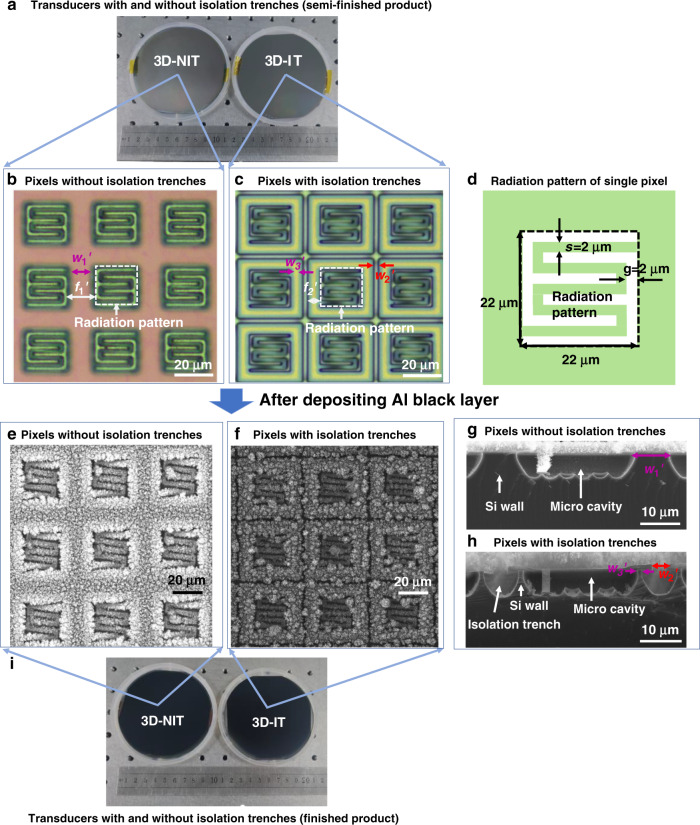
Photos, optical microscope images and SEM images of the two fabricated transducers. **a** Photo of the two transducers: 3D-NIT (left) and 3D-IT (right); optical microscope top view images of (**b**) 3D-NIT and (**c**) 3D-IT. **d** Schematic of the radiation pattern of a single pixel; SEM top view images of (**e**) 3D-NIT and (**f**) 3D-IT; SEM cross-sectional view images of (**g**) 3D-NIT and (**h**) 3D-IT. **i** Photo of the finished transducers: 3D-NIT (left) and 3D-IT (right)

Figure 5 summarizes the optical microscopy images and scanning electron microscopy (SEM) images of the 3D-IT and 3D-NIT transducers. Figure 5a shows a photograph of the two photothermal transducers before deposition of the Al black coating. An optical microscope was used to obtain top views of their microstructures, as shown in Fig. 5b, c. The radiation patterns of a single pixel were the same on both transducers. The side lengths of the radiation pattern were both 22 μm, and the double-S stripe width (*s*) and the gap widths (*g*) were both 2 μm, as shown in Fig. 5d. For 3D-NIT (Fig. 5b), the frame width (*f*_1_’) was 15 μm and the width (*w*_1_’) of the fabricated Si wall was approximately 11 μm because of isotropic Si etching. For 3D-IT (Fig. 5c), the frame width (*f*_2_’) was 6 μm, and the isolation trench had a width (*w*_2_’) of 3 μm, forming a much thinner Si wall with a width (*w*_3_’) of approximately 2 μm.

After the Al black layer was deposited, top-view images of the two transducers were acquired by SEM (Zeiss SUPRA 55 SAPPHIRE), as shown in Fig. 5e, f. 3D-IT exhibited physical isolation between adjacent pixels, while the connection with neighboring pixels remained in 3D-NIT. Cross-sectional SEM images of the two transducers are presented in Fig. 5g, h. The images proved that the two transducers formed Si microcavities under the pixel layer. The Si walls on the two transducers had narrow tops and wide bottoms due to isotropic Si etching. Figure 5i displays a photo of the two finished transducers.

### Measurement results

**Fig. 6:**
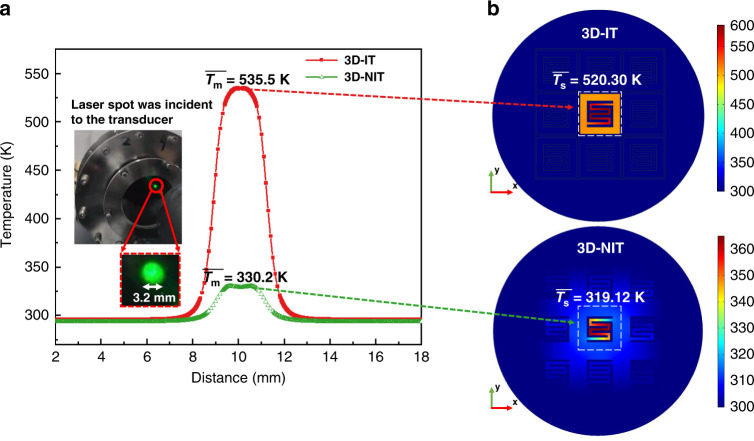
Measured and simulated stationary results. **a** Measured line temperature curves for 3D-IT and 3D-NIT. **b** Simulated average temperatures of 3D-IT and 3D-NIT in powered single pixels

In the measurement of the average temperature field, a 532 nm laser was focused and incident on the two fabricated infrared transducers. We used a thermal imager (InfraTec, VarioCAM HD head 680) to obtain a line temperature curve, as shown in Fig. 6a. The measured average temperature ($\bar T_m$) values in the powered areas of 3D-IT and 3D-NIT were 535.5 K and 330.2 K, respectively. The simulated average temperature ($\bar T_s$) values in powered single pixels of 3D-IT and 3D-NIT were 520.30 K and 319.12 K, as presented in Fig. 6b. The maximum difference of the measured and simulated average temperatures was below 15.2 K. The deviation may be caused by differences between the actual Si wall structure and the simplified simulated model. The actual Si walls had structures of narrow tops and wide bottoms, as shown in Fig. 5g, h. However, to reduce the computational memory and time needed, we set the silicon wall as a simplified cuboidal model (the top was as wide as the bottom), as shown in Fig. 3e, f. Another cause for the deviation may be the differences between the material parameters in the simulation model and the fabricated samples.

Radiation contrast is the ratio of the radiation power density of the highest and lowest apparent temperatures in the image. The formula for radiation power density is as follows:

3 $$E = \varepsilon \sigma T^4$$

According to Eq. (3), the radiation power density is proportional to the fourth power of the apparent temperature. Thus, the measured radiation contrast of transducer 3D-IT was 10.15 and that of transducer 3D-NIT was 1.47. The results proved that the introduction of isolation trenches not only increased the apparent temperature by 62% but also improved the radiation contrast by 590%.

**Fig. 7:**
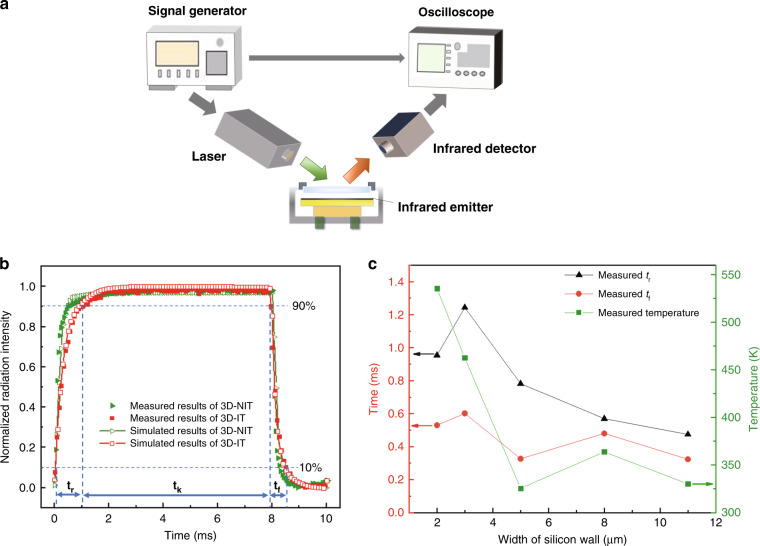
Experimental setup and measured and simulated results of transducers. **a** Experimental setup of the transient response for photothermal transducers. **b** Normalized radiation intensity of the measured and simulated time response curves for 3D-IT and 3D-NIT. **c** Measured *t*_r_, *t*_f_ and average stationary pixel temperature for photothermal transducers with different Si wall widths

The transient response determines the frame rate of the photothermal transducer. We used a laser signal controlled by a signal generator to measure the transient response characteristics of two fabricated transducers. The experiment setup is shown in Fig. 7a. The laser signal was incident to the pixels of the two transducers through a window of the vacuum chamber. The signal had a power density of 7.9 W/cm^2^, a period of 100 Hz and a duty cycle of 80%. The variation in the radiation intensity curves with time were measured utilizing an infrared point source detector (PVI-4TE-5, VIGO System S. A) and an oscilloscope (R&S RTB 2004, Germany). The given signal was also input to the oscilloscope for comparison.

Figure 7b displays the normalized radiation intensity of the measured and simulated transient response curves for the two transducers. As marked in Fig. 7b, the rising time (*t*_r_), falling time (*t*_f_), and holding time (*t*_k_) are defined as the time taken to rise from 10% to 90%, to fall from 90% to 10%, and to rise from 90% to 100% then fall to 90% of the maximum radiation intensity, respectively. *t*_r_ and *t*_f_ reflect the response speeds of the device. When *t*_r_ and *t*_f_ remain unchanged, *t*_k_ is determined by the holding time of the given signal. A shorter response time leads to a higher frame rate for the simulated infrared dynamic scenes^30^.

Table 1 summarizes the comparison of *t*_r_ and *t*_f_ based on the measured and simulated results. Compared to the state-of-the-art time response in a 2D infrared transducer (5.02 ms of *t*_r_ and 2.76 ms of *t*_f_)^23^, the proposed 3D transducer provided a much faster time response improvement of 428% for *t*_r_ and 421% for *t*_f_. However, there was still a certain deviation between the simulated and measured results, and the maximum percentage error was 21.9%. This maximum deviation occurred in the rapid falling time of 3D-NIT. From the perspective of measurement, the value of the falling time was so small (0.32 ms) that the noise and measurement error had a great impact on the measured value. From the perspective of simulation and fabrication, the error was mainly attributed to two aspects: the structural parameters and material parameters. First, the Si wall in the FEM model was not strictly consistent with the fabricated wall. The microstructures of the Si wall influenced the thermal resistance, so the simulated rising and falling times were inevitably different from the measured values. Second, as shown in Fig. 5e, f, although we kept the same thickness of Al black in the two transducers by controlling the deposition time, their Al black had different particle sizes since they were deposited on different substrates: one substrate without isolation trenches and the other substrate with isolation trenches. The Al black clusters in Fig. 5e were smaller than those in Fig. 5f, and this difference affected the time response of the transducers. However, we used the same Al black material parameters in the two simulated models. Therefore, the numerical results exhibited certain errors compared with the actual results. Moreover, for the 3D-IT transducer shown in Fig. 5f, some Al black clusters were so large that the neighboring pixels were partially connected, not completely isolated. Unfortunately, this could not be accurately included in our simulation model. Thus, the corresponding deviations occurred in the calculations.

**Table 1:** Comparison of the measured and simulated *t*_r_ and *t*_f_

Pixel pattern	M(*t*_r_)/ms	S(*t*_r_)/ms	Difference value/ms	Percentage error	M(*t*_f_)/ms	S(*t*_f_)/ms	Difference value/ms	Percentage error
3D-IT	0.95	0.83	−0.12	−12.6%	0.53	0.47	−0.06	−11.3%
3D-NIT	0.47	0.44	−0.03	−6.38%	0.32	0.39	0.07	21.9%

“M” stands for measured results and “S” for simulated results

In short, because the structural parameters and material parameters of the simulation models and fabricated transducers were not strictly consistent and undesired connections could occur in the preparation, it was difficult to avoid underestimation or overestimation. However, although our simulation model could not accurately predict the measurement results, it still provided a trend of thermal performance for the two transducers, and the maximum error was less than 21.9%. Therefore, it possessed advantages in predicting the thermal performance of photothermal transducers and providing qualitative comparisons between different structures. The calculation error could be further reduced by obtaining more accurate material and structural parameters in simulation models.

The thermal resistance determined the out-of-plane heat dissipation rate in the proposed 3D transducers. This key parameter was related to the width of the Si wall. We fabricated and measured photothermal transducers with different Si wall widths (*w’*) to observe the effect of thermal resistance on the stationary and transient thermal characteristics. SEM and optical microscope images of these photothermal transducers are shown in supplemental Section S4. *t*_r_, *t*_f_ and the average stationary pixel temperature are shown in Fig. 7c. Several trends could be found: (1) When *w’* exceeded 3 μm, *t*_r_ decreased as *w’* increased. This manifested in the response time of the photothermal transducer becoming shorter with decreasing thermal resistance. However, when *w’* changed from 2 μm to 3 μm, *t*_r_ exhibited an exceptional trend. For the case of 2 μm, the structure produced an acceptable *t*_r_ less than 1 ms. (2) *t*_f_ fluctuated from 0.4 ms to 0.6 ms when *w’* changed from 2 μm to 11 μm. The fluctuation range of *t*_f_ was quite limited. This implied that the frame rate of the photothermal transducer was more affected by *t*_r_ than *t*_f_. When *t*_r_ became shorter, the response speed became faster. (3) When *w’* changed from 2 μm to 5 μm, the average pixel temperature dropped significantly, from 535 K to 325 K. When *w’* changed from 5 μm to 11 μm, the average pixel temperature was maintained at a moderate level between 363 K and 325 K. In summary, these results demonstrated that when *w’* was 2 μm, the average pixel temperature was the most desirable, and the response speed was acceptable. The above experimental results indicated that it was challenging to simultaneously optimize the stationary and transient thermal characteristics. When designing 3D microstructures for photothermal transducers, we should balance the stationary and transient characteristics. For instance, if a high frame rate is more demanded, we can design a 3D microstructure with lower thermal resistance. In contrast, if a high stationary temperature is required, we can properly increase the thermal resistance. In addition, increasing the thermal resistance means a narrower Si wall, which increases the difficulty associated with the processing technology and deteriorates the mechanical stability of suspended pixels. Therefore, there is a tradeoff between the width of the Si wall and the mechanical stability of the transducer. Considering the above tradeoff issues, the thermal characteristics of the photothermal transducer can be flexibly controlled by designing different 3D microstructures in the silicon layer.

In conclusion, a thermal management approach of adjusting the nanoscale and microscale structures was proposed. The simulation and measured results showed that the stationary and transient properties could be significantly changed with thermal input and output modulation. Through systematic FEM simulations, the effects of in-plane patterns and out-of-plane structures on the stationary performance and transient response were explored. In general, the input thermal issues were determined by the black coating quality and the duty ratio of the in-plane patterns. The output thermal issues are attributed to the in-plane thermal resistance in the pixel pattern and the out-of-plane thermal resistance in the Si layer. Compared to 2D transducers, the proposed 3D transducer with an isolation trench (3D-IT) exhibited overwhelming advantages in thermal crosstalk, temperature difference, time response and mechanical stability. Compared to the 3D transducers without isolation trench (3D-NIT), the proposed 3D-IT transducer exhibited superior crosstalk prevention and temperature difference, as well as compatible time response and mechanical stability. The proposed photothermal transducer achieved 2.2 K × 2.2 K pixels on a 4-inch Si substrate. If the processing technology permitted, a pixel array beyond 3.8 K × 3.8 K could be obtained by 8-inch silicon micromachining. In particular, the proposed manufacturing method was quite simple, low-cost and had great potential for achieving a much larger pixel scale provided that larger Si processing (such as 12-inch or 16-inch processing) was guaranteed. The proposed nano- and microthermal modulation strategy provided guidance not only for thermal emitters but also for other thermal management issues, such as IC design, energy harvesting and thermal stealth.

## Supplementary information

Demonstration of thermal modulation using nanoscale and microscale structures for ultra-large-pixel-array photothermal transducers
