# Supplementary material for: Demonstration of thermal modulation using nanoscale and microscale structures for ultralarge pixel array photothermal transducers
Source: Microsyst Nanoeng. 2021 Dec 3;7:102. doi: 10.1038/s41378-021-00315-5 (PMC8642404; doi:10.1038/s41378-021-00315-5)
Supplement: Supplementary file 1 — Demonstration of thermal modulation using nanoscale and microscale structures for ultra-large-pixel-array photothermal transducers [file 41378_2021_315_MOESM1_ESM.docx]

**Demonstration of thermal modulation using nanoscale and microscale structures for ultra-large-pixel-array photothermal transducers**

*Jinying Zhang^1#*^, Defang Li^1#^, Zhuo Li^1*^, Xin Wang^1^ and Suhui Yang^1^*

^1^ Beijing Key Laboratory for Precision Optoelectronic Measurement Instrument and Technology, School of Optics and Photonics, Beijing Institute of Technology, Beijing, 100081, P. R. China

^#^Co-first author

*Co-corresponding author: [jyzhang@bit.edu.cn](mailto:jyzhang@bit.edu.cn); lizhuo@bit.edu.cn

**Supplemental Section S1: SEM images and absorption characteristics of Al black coating**


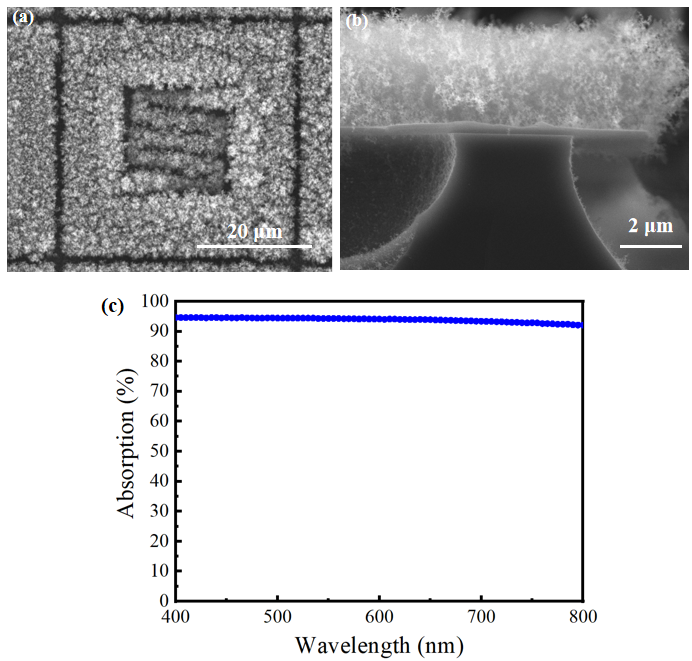


**Fig. S1 SEM images and measured absorption spectrum of Al black coating.** (a) SEM image of Al black coating top view; (b) SEM image of Al black coating cross-sectional view. (c) measured absorption spectrum of Al black coating.


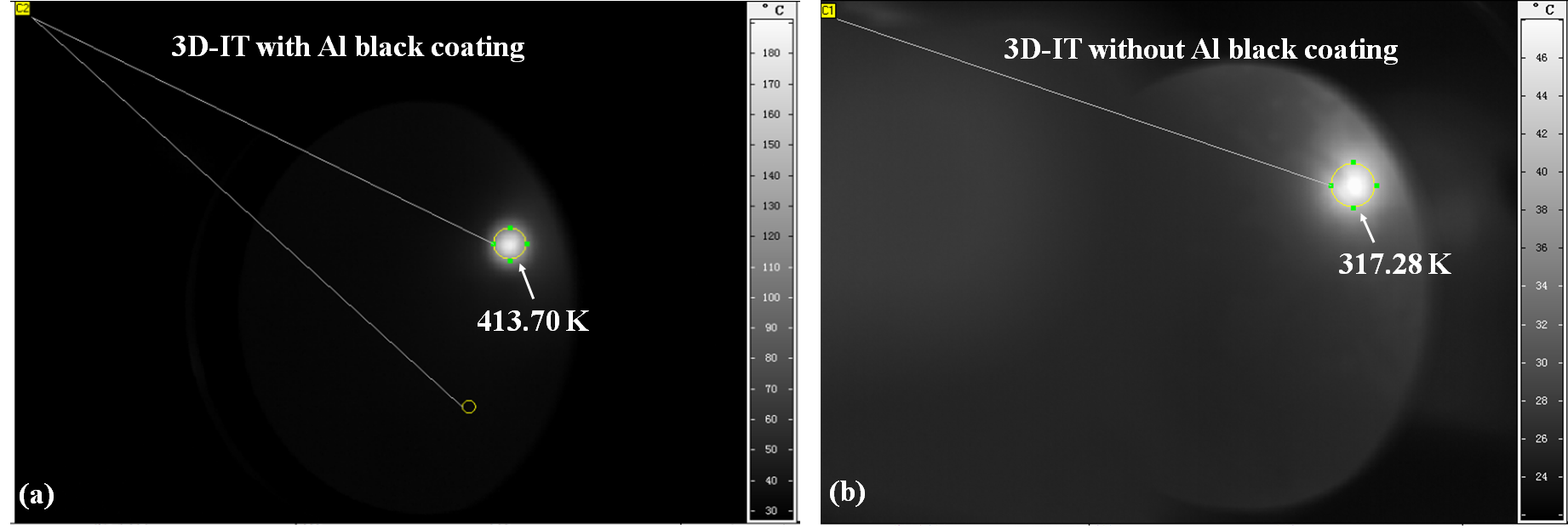


**Fig. S2 Thermal images of 3D-IT transducer with and without Al black coating.** (a) 3D-IT with Al black coating; (b) 3D-IT without Al black coating.

Al black coating with nanoscale structures contributed to an increase by 5.6 folds (∆T_1_=413.70-300=113.70 K, ∆T_2_=317.28-300=17.28 K) in the temperature difference compared to the one without Al black coating.

**Supplemental Section S2: Parameter setting for COMSOL Finite Element Simulations**

The material and structural parameters in the three models (2D photothermal transducer and 3D photothermal transducers with and without isolation trenches). The thickness values of PI layer and Al black coating were obtained by SEM measurement. Considering the thickness of Si wafer had no remarkable effect on the simulation results, the silicon thickness was set as 6 μm to reduce the heavy computational burden. Moreover, the Cr layer had a quite small thickness (around 5 nm) so that its effect on thermal performance could be neglected. The material parameters of Si layer and PI layer were set according to the bulk materials and reference^1-3^. The thermal conductivity of Al black layer was measured by heat conduction coefficient measurement device (TC3000E) and its constant pressure heat capacity was measured by differential scanning calorimeter (NETZSCH, DSC214).

**Table S1: Material and structural parameters in FEM models**

| Parameter | Si | PI | Al black |
| --- | --- | --- | --- |
| Thickness (μm) | 6 | 0.28 | 4 |
| Density (kg/m^3^) | 2329 | 1350 | 54 |
| Constant pressure heat capacity (J/ (kg ∙ K)) | 700 | 650 | *C** |
| Thermal conductivity (W/ (m ∙ K)) | 130 | 0.2 | *K** |

The measured thermal conductivity *C** and constant pressure heat capacity *K** of Al black coating was illustrated in Fig. S3.


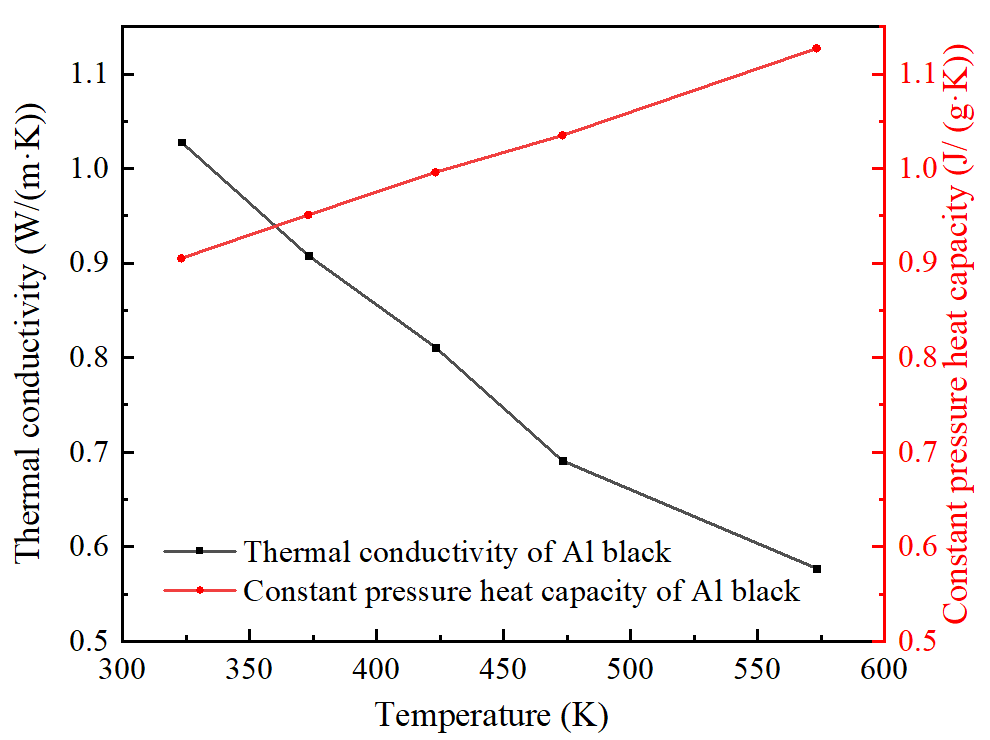


**Fig. S3 Measured thermal conductivity and constant pressure heat capacity of Al black coating.**

**Supplemental Section S3: Fabrication process of photothermal transducer**


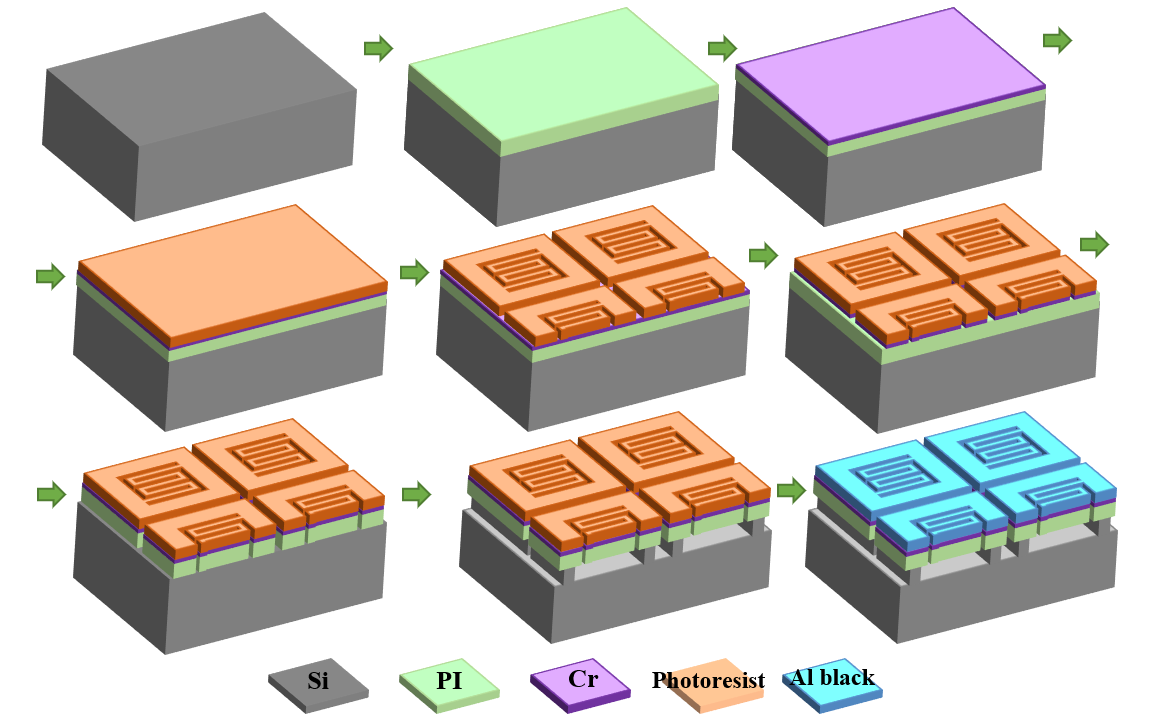


**Fig. S4 The fabrication process of the photothermal transducer.**

The fabrication process of photothermal transducer is as below.

1. A Si wafer with polished side was cleaned and ready for use.

2. A PI layer (around 280 nm thick) was spin-coated on the polished side of the Si wafer. After that, the PI layer would go through a stepped heating process.

3. A Cr layer with 5 nm thickness was deposited on the PI layer.

4. A photoresist layer was spin-coated on the Cr layer.

5. The photoresist layer was patterned by UV-exposure and development and formed the pixel arrays pattern.

6. Using the photoresist layer as an etching mask, the Cr layer was patterned by wet-etching. The Cr layer kept the same pattern as the photoresist layer by precisely controlling the etching time.

7. The PI layer was patterned by dry-etching and formed the same pattern as Cr layer and photoresist layer.

8. Utilizing the isotropic etching, the Si layer was etched to form a number of silicon microcavities and micro isolation trenches.

9. Finally, the rest photoresist was removed through immersing it into the acetone and cleaning by deionized water and then the Al black coating was deposited on the Cr layer by thermal evaporation.

After above procedures, the transducer would be placed in an oven with a temperature of 300°C for more than 30 minutes. This annealing step helped the transducer release its residual stress. Therefore, it would not have a large deformation when the working temperature was less than the annealing temperature.

**Supplemental Section S4: SEM and optical microscope images for photothermal transducers with different Si wall widths**


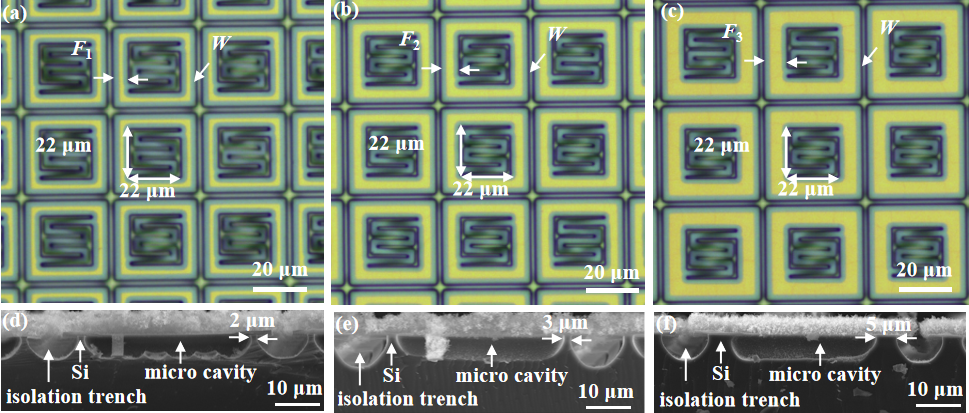


**Fig. S5 The optical microscope images and SEM images of the fabricated pixel structures with different Si wall widths.** (a-c) optical microscope images of the three fabricated transducers with the same frame gap (W = 3 μm) before the preparation of Al black. (a) frame width F_1_ was 6 μm and Si wall width was 2 μm. (b) frame width F_2_ was 7 μm and Si wall width was 3 μm. (c) frame width F_3_ was 9 μm and Si wall width was 5 μm. (d-f) SEM images of the three fabricated transducers in cross-sectional view: (d) frame width F_1_ was 6 μm and Si wall width was 2 μm. (e) frame width F_2_ was 7 μm and Si wall width was 3 μm. (f) frame width F_3_ was 9 μm and Si wall width was 5 μm.

**Supplemental Section S5: Images of photothermal transducer and experimental setup**


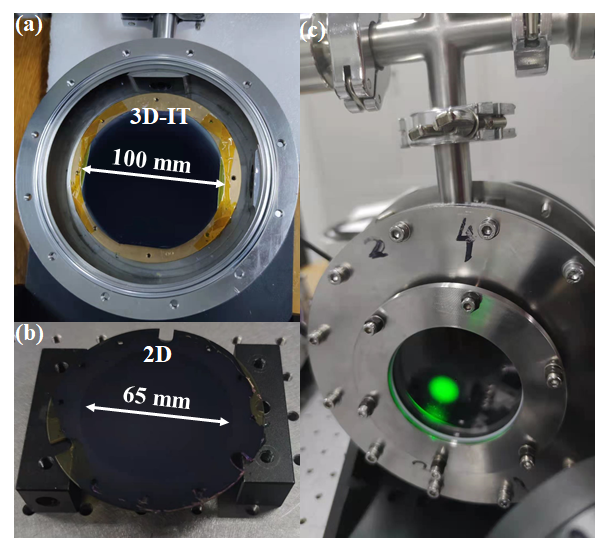


**Fig. S6 Images of the photothermal transducers and vacuum chamber.** (a) 3D-IT photothermal transducer with effective diameter of 100 mm installed in the vacuum chamber. (b) 2D photothermal transducer with effective diameter of 65 mm. (c) A 532 nm laser was incident on the photothermal transducer through the chamber window. The vacuum chamber was connected to a water-cooling system and a vacuum system.

Supplemental References

1 Ghodssi, R. & Lin, P. J. C. I. T. MEMS Materials and Processes Handbook. **78**, 1480–1482 (2011).

2 Wang, X., Zhao, Q., Li, Z., Yang, S. H. & Zhang, J. Y. Measurement of the thermophysical properties of self-suspended thin films based on steady-state thermography. *Opt. Express* **28**, 14560-14572, doi:10.1364/oe.392198 (2020).

3 Haynes, W. M. J. C. P. CRC Handbook of Chemistry and Physics, 95th Edition. **257**, 423 (2016).
